# Novel Lipopolysaccharide-Responsive Vesicle Trafficking, Beach- and Anchor-Containing (LRBA) Gene Mutation Identified in a Pediatric Patient: A Case Report

**DOI:** 10.7759/cureus.65434

**Published:** 2024-07-26

**Authors:** Jasleen Dua, Renuka Jadhav, Vineeta Pande, Mridu Bahal, Shailaja V Mane

**Affiliations:** 1 Pediatrics, Dr. D. Y. Patil Medical College, Hospital and Research Centre, Dr. D. Y. Patil Vidyapeeth, Pune, IND

**Keywords:** hypogammaglobinemia, rare genetic disorder, autosomal recessive disorders, primary immunodeficiency disease, lrba deficiency

## Abstract

Homozygous mutations in the lipopolysaccharide-responsive vesicle trafficking, beach- and anchor-containing (*LRBA*) gene lead to a syndrome characterized by early-onset hypogammaglobulinemia, autoimmunity, lymphoproliferation, and inflammatory bowel disease. This report describes a 10-year-old female who experienced three seizure episodes, including two generalized tonic-clonic seizures (GTCS) and one focal seizure, alongside septic shock. The patient had a history of recurrent respiratory tract infections, inflammatory bowel disease, multiple blood transfusions, lymphadenopathy, significant organomegaly, and hematological abnormalities, all consistent with an *LRBA* deficiency. This case highlights the critical need for prompt recognition and identification of *LRBA* gene mutations to enable timely management and improve patient outcomes.

## Introduction

Lipopolysaccharide-responsive beige-like anchor protein (*LRBA*) deficiency is caused by mutations in the *LRBA *gene. *LRBA *belongs to the pleckstrin homology-beige and Chediak-Higashi-tryptophan-aspartic acid dipeptide (PH-BEACH-WD40) protein family. Although the function of *LRBA *is not fully understood, it is known to co-localize with cytotoxic T-lymphocyte-associated protein 4 (*CTLA4*) within recycling endosomes in normal T cells, suggesting a role in the regulation of these endosomes. Homozygous mutations in *LRBA *result in the loss of *LRBA *protein expression.

Primary immunodeficiencies (PIDs) constitute a spectrum of genetically determined disorders characterized by varying degrees of immune system dysfunction [1]. Among these, lipopolysaccharide-responsive beige-like anchor protein (*LRBA*) deficiency is one such condition within this group, characterized by an autosomal recessive inheritance pattern stemming from mutations in the *LRBA *gene [2]. Individuals with *LRBA* deficiency exhibit a wide range of clinical symptoms, including autoimmune cytopenia, hypogammaglobulinemia, and recurrent infections, which underscore the critical role of *LRBA *in immune system regulation and function [3].

*LRBA *plays a pivotal role in the immune response, regulating the function and trafficking of *CTLA4*. *CTLA4 *acts as an inhibitory checkpoint, maintaining immune homeostasis by downregulating immune responses [4]. *LRBA *binds to the cytoplasmic tail of *CTLA4*, thereby modulating its degradation process. By preventing the trafficking of *CTLA4* to lysosomes for degradation, *LRBA *protects *CTLA4 *and ensures its proper function in immune regulation [5]. Dysregulation or deficiency of *LRBA *disrupts this regulatory mechanism, leading to aberrant immune responses and the clinical manifestations observed in *LRBA *deficiency [4]. Elucidating the intricate interplay between *LRBA *and *CTLA4 *provides insights into the pathophysiology of *LRBA *deficiency and identifies potential targets for therapeutic interventions aimed at restoring immune homeostasis in affected individuals.

## Case presentation

We report the case of a 10-year-old female, firstborn of third-degree consanguineous parents, delivered vaginally at term with no need of NICU admission, weighing 3 kg. The patient experienced two episodes of generalized tonic-clonic seizures, each lasting for one minute, characterized by the uprolling of her eyeballs. Additionally, she had one episode of a focal seizure, which involved deviation of her mouth to the left side, uprolling of her eyes, and frothing from her mouth, lasting for 15 minutes, necessitating intubation due to a Glasgow Coma Scale score of 6/15. Concurrently, the patient exhibited persistent ear discharge and signs of septic shock, prompting transfer to the pediatric intensive care unit (PICU) for management with inotropes, anti-seizure medications, anti-edema therapies, and broad-spectrum antibiotics.

**Figure 1:**
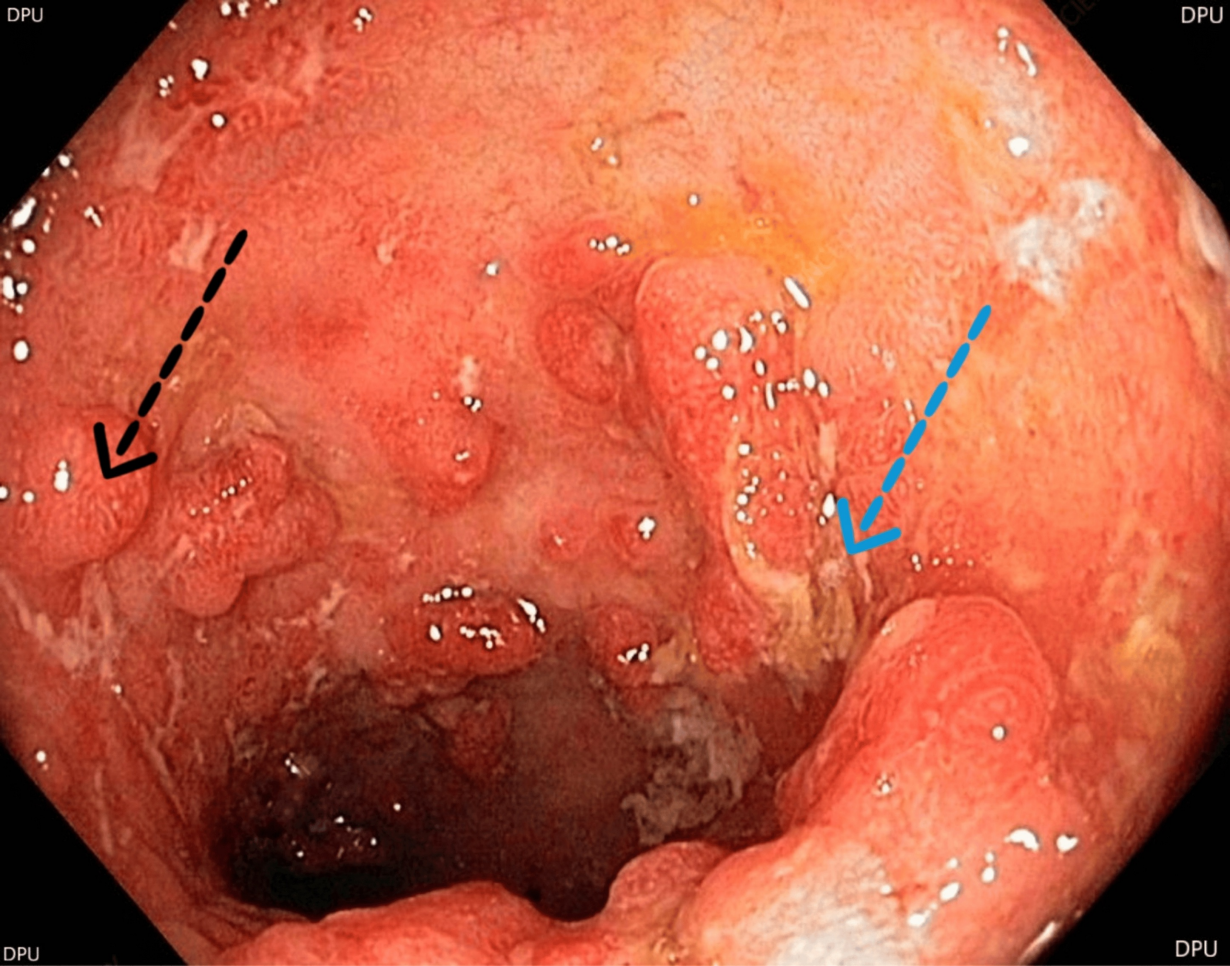
Colonoscopy report Blue arrow - ulcer with surrounding erythematous mucosa Black arrow - pseudopolyp

Her medical history was notable for recurrent respiratory tract infections, frequent diarrheal illnesses, three episodes of otitis media accompanied by pus discharge from the ear, multiple blood transfusions, and prior treatment for immune thrombocytopenic purpura at age 4. A colonoscopy at age 8 revealed severe ulcerative colitis characterized by multiple deep ulcers with erythematous mucosa and slough-covered lesions in the ileum, extending proximally from the anorectal junction to the ileocecal valve (Figure 1).

Clinical examination upon admission indicated high-grade fever, tachycardia, tachypnea, bounding pulses, flash capillary refill time, hypotension, and enlarged cervical, axillary, and inguinal lymph nodes. Abdominal examination revealed hepatomegaly and splenomegaly, and anthropometric measurements showed height and weight below the third percentile.

Laboratory investigations demonstrated pancytopenia accompanied by spherocytosis on the peripheral blood smear, along with elevated C-reactive protein (CRP) levels (Table 1).

**Table 1:** Blood investigations CRP, C-reactive protein; SGOT, serum glutamic oxaloacetic transaminase; SGPT, serum glutamic pyruvic transaminase; TLC, total leucocyte count

Parameter	Observed value	Reference range
Hemoglobin	9.9	12-14.5 g/dL
TLC	3,800	4,000-10,800 /µL
Platelet count	70,000	150,000-410,000 /µL
Neutrophils	78	40-80 %
Lymphocytes	13	20-40 %
Total bilirubin	0.5	0.22-1.20 mg/dL
Bilirubin - conjugated	0.3	<0.5 mg/dL
Bilirubin - unconjugated	0.2	0.1 to 1.0 mg/dL
SGPT	21	7 to 45 U/L
SGOT	186	8-50 U/L
Blood urea	19	17-49 mg/dL
Serum creatinine	0.44	0.26-0.61 mg/dL
Sodium	143	138-145 mmol/L
Potassium	5	3.50-5.10 mmol/L
Chloride	112	98-107 mmol/L
CRP	314	<10 mg/L

Non-reactive serology and cerebrospinal fluid analysis (Table 2) indicated bacterial meningitis with *Streptococcus pneumoniae* isolated from cultures.

**Table 2:** CSF routine and microscopic examination CSF, cerebrospinal fluid; TLC, total leucocyte count

Parameter	Observed value	Reference range
Glucose	36	40-80 mg/dL
Protein	79.7	15-45 mg/dL
TLC	800	0-10 /cu mm
Lymphocytes/polymorphs	5%/75 %	Predominantly lymphocytes

Pseudomonas aeruginosa was cultured from ear discharge, indicating secondary infection. Two-dimensional echocardiography and abdominal/pelvic ultrasonography were unremarkable.

Electroencephalography demonstrated abnormal background asymmetry over the right hemisphere and periodic lateralized epileptiform discharges. Immunoglobulin levels suggested hypogammaglobulinemia (Table 3).

**Table 3:** Immunoglobulin panel IgA, immunoglobulin A; IgG, immunoglobulin G; IgM, immunoglobulin M

Parameter	Observed value	Reference range
IgG	203	650-1,452 mg/dL
IgM	688	50-240 mg/dL
IgA	99.6	44-252 mg/dL

Given the findings of hypogammaglobulinemia, PID was suspected, and subsequent genetic testing identified a heterozygous mutation in the *LRBA *gene (*c.47_57delATGACGGGGGA*, p. Asp16Glyts*10), consistent with an autosomal recessive inheritance pattern (Table 4).

**Table 4:** Whole exome sequencing report Test result: probable compound heterozygous, likely pathogenic, and variant of uncertain significance identified

Gene and transcript	Location	Variant	Zygosity	Classification	Disease	Inheritance
LRBA (NM_ 006726.4)	Exon 23	c.3294T>G (P. Asp1098Glu)	Heterozygous	Uncertain significance	Immunodeficiency. Common variable, 8, with autoimmunity.	Autosomal recessive
LRBA (NM_ 006726.4)	Exon 2	C.47_57 delATGACGGGGGA (p.Asp16Glyfs*10)	Heterozygous	Likely pathogenic	Immunodeficiency. Common variable, 8, with autoimmunity.	Autosomal recessive

The patient was discharged on co-trimoxazole prophylaxis and sirolimus (an immunosuppressant) and is on regular follow-up due to recurrent respiratory and gastrointestinal infections, as well as otitis media. She has had two hospital admissions, each lasting three and four days, respectively. Hematopoietic stem cell transplantation (HSCT) was recommended during follow-up due to her recurrent infections, but it was unaffordable for the family.

## Discussion

Immune dysregulation associated with *LRBA* deficiency commonly presents as enteropathy, autoimmune cytopenia, granulomatous-lymphocytic interstitial lung disease, and lymphadenopathy [6,7]. Less frequently observed symptoms include cerebral granulomas, type 1 diabetes mellitus, idiopathic thrombocytopenic purpura (ITP), alopecia, uveitis, myasthenia gravis, and eczema [8]. These manifestations indicate underlying inflammatory processes or organ dysfunction, complicating clinical management and necessitating comprehensive therapeutic approaches.

The *LRBA *deficiency has also been linked in the literature to various other disorders such as neonatal diabetes, polyarthritis, and early-onset inflammatory bowel disease [9-11]. Recurrent infections are a prominent feature, reflecting compromised immune function and susceptibility to microbial pathogens [9]. These infections can involve multiple organ systems, adding to the complexity of the condition and potentially leading to chronic complications if not effectively treated. Furthermore, diminished regulatory T (Treg) and B cell counts underscore specific immunological impairments associated with *LRBA *deficiency [1].

Identification of the *LRBA *gene mutation offers significant insights into the molecular mechanisms driving clinical manifestations, enabling targeted therapeutic interventions and informed genetic counseling. Several studies have shown that abatacept-mediated targeted therapy and HSCT are effective treatments for patients with *LRBA *deficiency. Abatacept is a fusion protein that combines the extracellular domain of human cytotoxic T-lymphocyte-associated antigen 4 with a modified Fc region of human IgG1, modulating immune responses by interfering with T cell activation, thereby alleviating the symptoms. HSCT offers a potential cure by replacing the defective immune system with healthy donor stem cells. These therapeutic approaches have shown promising results in clinical studies, significantly improving patient outcomes and offering hope for long-term disease management and quality of life enhancement for individuals with *LRBA *gene mutation [12-14]. This holistic approach emphasizes the integration of clinical assessment with advanced molecular techniques to pinpoint genetic mutations and tailor personalized management strategies for complex medical conditions.

## Conclusions

This case highlights the clinical challenges and complexities associated with *LRBA *deficiency. The patient's recurrent infections, including respiratory, gastrointestinal, and otitis media, underscore the importance of early diagnosis and consistent follow-up in managing such immunodeficiencies. Despite treatment with co-trimoxazole prophylaxis and sirolimus, the frequency and severity of infections necessitated consideration of HSCT, which was ultimately unaffordable. This case emphasizes the need for accessible treatment options and supports the role of HSCT in providing a potential cure for patients with *LRBA *deficiency. Future advancements in treatment accessibility and early intervention strategies are crucial for improving outcomes in similar cases.
